# *Salmonella* in Pig Farms and on Pig Meat in Suriname

**DOI:** 10.3390/antibiotics10121495

**Published:** 2021-12-06

**Authors:** Patrick Butaye, Iona Halliday-Simmonds, Astrid Van Sauers

**Affiliations:** 1 Department of Biosciences, School of Veterinary Medicine, Ross University, Basseterre 00334, Saint Kitts and Nevis; IHalliday-Simmonds@rossvet.edu.kn; 2 Department of Pathobiology, Pharmacology and Zoological Medicine, Faculty of Veterinary Medicine, Ghent University, B-9820 Merelbeke, Belgium; 3 The Veterinary Services, Ministry of Agriculture, Paramaribo, Suriname; astrid.vansauers@gmail.com

**Keywords:** *Salmonella enterica*, pigs, Suriname, whole genome sequencing

## Abstract

*Salmonella* is one of the most important food borne zoonotic pathogens. While mainly associated with poultry, it has also been associated with pigs. Compared to the high-income countries, there is much less known on the prevalence of *Salmonella* in low- and middle-income countries, especially in the Caribbean area. Therefore, we investigated the prevalence of *Salmonella* in pigs and pig meat in Suriname. A total of 53 farms and 53 meat samples were included, and *Salmonella* was isolated using standard protocols. Strains were subjected to whole genome sequencing. No *Salmonella* was found on pig meat. Five farms were found to be positive for *Salmonella*, and a total of eight different strains were obtained. Serotypes were *S.* Anatum (*n* = 1), *S.* Ohio (*n* = 2), a monophasic variant of *S.* Typhimurium (*n* = 3), one *S.* Brandenburg, and one *S.* Javaniana. The monophasic variant of *S.* Typhimurium belonged to the ST34 pandemic clone, and the three strains were very similar. A few resistance genes, located on mobile genetic elements, were found. Several plasmids were detected, though only one was carrying resistance genes. This is the first study on the prevalence of *Salmonella* in pigs in the Caribbean and that used whole genome sequencing for characterization. The strains were rather susceptible. Local comparison of similar serotypes showed a mainly clonal spread of certain serotypes.

## 1. Introduction

Few studies have been performed on *Salmonella* in the Caribbean region, and those mainly dealt with the prevalence in poultry. Prevalences were very variable depending on the study. Moreover, most studies were performed in Trinidad and were mainly on food products, both fresh and ready to eat. The prevalence on those products varied between 0% (mainly ready to eat) and 7%. Several serotypes were isolated, including *S.* Agona *S.* Kiambu, *S.* Kentucky, *S.* Derby, and *S.* Mbandaka as the most reported. The few studies in the Caribbean that have been performed on live animals dealt with layer chickens, and apart from one study, all were on eggs. Eggs have been a major focus for the Caribbean. Prevalence on eggs varied from 1 to 13%, and a multicountry study in 2014 showed that 3% of the layer farms were positive. Prevalence in Barbados on layer farms was the highest, with 73% of the farms being positive. It should be noted that for the different studies, different sampling and isolation methods were used, and as such, comparisons are difficult [1,2].

Several studies were performed on pet animals and mainly wildlife. In dogs, eight *Salmonella* strains were recovered [3]; in iguanas, mongooses, tree boa, leatherback turtles, blue land crabs, and toads, a few strains were found, though it should be noted that the serotypes were very different, and that the iguanas had to be regarded as wildlife. These strains in general carried few resistances [4,5,6,7,8,9,10].

There are very few studies on pork or pigs in the Caribbean region. An old study in Trinidad and Tobago showed that about 18% of the swine carcasses sampled were positive for *Salmonella* [11], while a small-scale baseline study in the Bahamas showed that 8/42 of the retail meat samples were positive for *Salmonella*. At a pig slaughter plant, 44.1% (15/34) and 2.9% (1/34) were found positive at the beginning and the end of the slaughter process, respectively [12]. In both diarrheic and non-diarrheic pigs of different ages from Trinidad, a prevalence between 3 and 4.5% was recorded. A more recent study in Cuba demonstrated a prevalence of 2.2% in weaned piglets [13]. Apart from those studies, we found no other studies in live pigs.

In humans, there is no systematic surveillance of nontyphoid *Salmonella* infections, and as such, the incidence is unknown. However, the incidence has been estimated at an annual rate of over five cases per 100,000 persons in Trinidad and Tobago [6,14].

Pig production is important in the food supply in Suriname. In 2018, 208 farms were active, with a total of 21,362 pigs divided over the different farms. Since pig meat can also be a major source of *Salmonella* infections in humans, we investigated the prevalence, as well as the types of *Salmonella* in pigs in Suriname.

## 2. Materials and Methods

### 2.1. Sampling

Samples from pigs were taken at the only pork slaughterhouse in Paramaribo, Suriname in 2018. Farms were located in the districts of Paramaribo, Wanica Saramacca, and Coronie. Pigs were mixed breed pigs, with breeds involved being the Dutch Landrace, Yorkshire, Pietrain, and Duroc. Pigs originated from 53 farms in the central part of Suriname that were delivering pork to the slaughterhouse. One pig per farm was sampled except for two farms; from one of these, two pigs were sampled on the same day; and from another farm, two pigs were sampled, but on different sampling days. A sample was taken from the cecum and ileocecal lymph node of each pig.

Meat samples were obtained from 53 retail stores in Paramaribo and Wanica. Most retail stores are in Paramaribo, which contains about half of the population of Suriname. Per retail store, one piece of fresh pork chop sample was obtained. There is no food tracing system in Surname, and as such, the slaughter date nor origin of the meats could be traced.

### 2.2. Isolation and Identification

*Salmonella* was isolated using the ISO 6579 annex D. Briefly, after homogenization, the sample (25 g) was inoculated in Buffered Peptone Water (BPW, Bio-Rad, Hercules, CA, USA) (1:10, W/V) and incubated for 16–20 h at 37 °C. Of this, 0.1 mL was inoculated on a Modified Semisolid Rappaport Vassiliadis agar plate (MSRV, Bio-Rad, Hercules, CA, USA) and incubated for 21–27 h at 41 °C. Positive samples were inoculated on Xylose Lysine Deoxycholaat agar plate (XLD, Bio-Rad, Hercules, CA, USA) and a nalidixine-BGA plate and incubated for 21–27 h at 37 °C. Suspected colonies were inoculated on Triple Sugar Iron agar (TSI, Bio-Rad, Hercules, CA, USA) and lysine decarboxylase broth (Thermo Fisher Scientific, Waltham, MA, USA) and incubated for 18–24 h at 37 °C for presumptive identification. Further identification was performed using the Gram-Negative ID Plate (Sensititre GNID, TREK Diagnostic Systems, Cleveland, OH, USA), using the protocols provided by the supplier, and using a Sensititre OptiRead analyzer (TREK Diagnostic Systems, Cleveland, OH, USA). *Salmonella* isolates were then serogrouped using the Wellcolex Color *Salmonella* Rapid Latex Agglutination Test Kit (Thermo Fisher Scientific, Waltham, MA, USA). Strains that could serogrouped were selected for whole genome sequencing.

### 2.3. Whole Genome Sequencing

Purified strains were sent to Macrogen (Seoul, Korea) for DNA extraction and sequencing on an Illumina platform using a TruSeq Babi DNA kit and 151 bp long paired-end sequencing.

Raw sequences (fastq files) were submitted to the NCBI database under PRJNA751882 with SAMN20584910, SAMN20584911, SAMN20584912, SAMN20584913. SAMN20584914, SAMN20584915, SAMN20584916, and SAMN20584917.

Sequences were trimmed and assembled using SKESA [15]. Annotation was done with PROKKA [16] and RAST [17]. The serotype was determined with Sistr [18]. The MLST profile was determined using ‘mlst’ [19]. Phylogenetic analysis of the isolates of a same serotype was done with NASP [20]. Further analysis of the strains was done using ARIBA against the plasmid finder [21], ARg-ANNOT [22], and ResFinder [23,24]. Plasmids were confirmed with Platon [25]. Using RAST analysis, we located the different associated genes.

### 2.4. Statistical Analysis

Confidence intervals of the prevalence of *Salmonella* on farms in Suriname were calculated using exact binomials in an Excel file.

## 3. Results 

### 3.1. Prevalence, Serotyes, and Epidemiology

*Salmonella* could not be isolated from any of the food samples. Of the 53 farms investigated, a total of eight strains were isolated. Strains originated from five different farms (9%, of the farms were positive, confidence interval 3.1–21%) and seven different animals. Five different serotypes were isolated, including one *S.* Anatum, *S.* Ohio (*n* = 2), a monophasic variant of *S.* Typhimurium (*n* = 3), one *S.* Brandenburg, and one *S.* Javaniana (Table 1).

### 3.2. Antimicrobial Resistance Genes

All strains had at least one resistance gene (Table 2), though it should be taken into account that all *Salmonella* have the *aac(6′)* gene in their chromosome [26]. As such, three strains did not show any additional resistance gene. A total of five out of eight strains were resistant to penicillins, mediated by the *bla*_TEM-1_ gene. All strains had at least one resistance gene for aminoglycosides, and some strains had up to four different aminoglycoside resistance genes. Four strains carried the *sul2* gene, indicating sulfonamide resistance. Only two strains carried the tetracycline resistance gene, *tet*(B), and one strain carried a trimethoprim resistance gene, *dfrAB*. However, the latter strain was susceptible to sulfonamide.

### 3.3. Plasmids

Two strains did not carry any plasmids (strain 174, *S.* Ohio; and 250, *S.* Javaniana). Five different Inc plasmids were detected: IncQ1_1, IncI1_1_Alpha, IncFIA(HI1)_1_HI1, IncH1A/B, and IncHI1B(R27)_1_R27.

Three different colicin plasmids were found. The full sequence of a small plasmid ColpVC_1 was found by Platon in strain 173, and this plasmid was nearly similar to earlier described plasmids in *E. coli* and *Salmonella* (NCBI BLAST result). The Col440I_1 plasmid from the *S.* Brandenburg and *S.* Ohio strain were identical. It was a small circular plasmid of 1748 bp and contained four genes. The ColRNAI_1 plasmid from the *S.* Brandenburg strain was 4597 bp and had seven genes.

### 3.4. Association of Resistance Genes with Mobile Genetic Elements

The *aph(6)-Id (strB), aph(3″)-Ib (strA)* genes in the three monophasic *S.* Typhimurium strains were located on a same contig together with a *sul2*. However, there were no other genes on that contig, making its location speculative. In the *S.* Brandenburg strain, this same gene cluster was located on a 63,539 pb contig that also contained the *bla*_TEM-1_ gene, downstream of the *sul2* gene. In the *S.* Ohio strain 543, the *aph(6)-Id, aph(3″)-Ib*, *bla*_TEM-1_, and *sul2* genes were located as one cluster with a similar structure to the *S.* Typhimurium and *S.* Brandenburg strains, and associated with the IS*1* gene *InsB*, though the downstream part associated with Tn*7* was missing on the contig. In addition, copper and zinc resistance genes were present in this cluster. This gene cluster most likely was associated with a mobile Tn*7*-like transposon.

The *aph(3′)-Ia* gene was typically located on the InQ1_1 plasmid in the three monophasic *S.* Typhimurium strains.

The tetracycline resistance genes found in the strains of this collection were located on the same structure; however, the contig containing the *tet*(B) gene was too small to determine the exact location Blast analysis of the whole contig showed that it was similar to a chromosomal location in the bacterial species *Glasseralla parasuis*, *E. coli*, and *Shigella flexneri*.

## 4. Discussion

This is one of the few more systematic investigations on *Salmonella* in pigs in the Caribbean. Though few samples per farm could be tested, when lowering the sensitivity for detecting positive farms, we found 5/53 farms positive, which was close to 10% of the farms being positive. This was higher than what has been found in Cuba, though it should be noted that this study was conducted on weaned pigs, while our study was conducted on pigs at slaughter [13]. Our findings were lower than what was found on pig carcasses in Trinidad in 1978, while higher than what was found in the same country in fecal material of pigs in 1993 (4.1% of the 294 pigs) and in 1994, with 4.5% of the diarrheic pigs and 3.4% (*n* = 179) of the non-diarrheic pigs (*n* = 117) originating from 25 farms sampled at different ages being positive [1,27]. The variations can also be explained by the age or carcass contamination, as it is known that older pigs are in general more prone to be positive than piglets [28], while contamination at the slaughter line may increase the apparent prevalence [29].

In this study, we found five different serotypes. The most commonly found in the European Union and the US are *S.* Typhimurium, *S.* 1,4,[5],12:i:-, *S.* Derby, and *S.* Rissen, though this varies in time and space [29,30]. While the monophasic variant of *S.* Typhimurium is one of the most commonly found serotypes in pigs worldwide, and *S.* Brandenburg is less commonly found, the other serotypes, *S.* Ohio, *S.* Anatum, and *S.* Javaniana, are more rarely reported. This may indicate that there may be some differences in the epidemiology of Salmonella in the Caribbean; however, due to the low numbers of strains recovered, this should be interpreted with care. Two of the monophasic *S.* Typhimurium strains were the same based on the NASP SNP analysis, as they had no SNPs in the core genome, while the other strain had only 235 SNPs different. It was striking that the two equal monophasic *S.* Typhimurium strains were from two different farms. Unfortunately, we could not determine whether those two farms had epidemiological connections. The two different monophasic *S.* Typhimurium strains came from the same farm, but from different pigs, indicating several different clones were circulating in that farm. All the monophasic Typhimurium strains were ST 34, an epidemic clone in Europe [31] and other parts of the world that is to be regarded as a pandemic strain [32,33,34]. Compared to other *S.* Typhimurium ST34 strains, the Caribbean clone was remarkably susceptible. This may be due to the limited availability of antimicrobials in Suriname. *S.* Anatum ST64 has been reported as one of the most prevalent strains on retail pork in Juiangsu China [35]; however, few data are available on this clone. The two *S.* Ohio strains originated from different farms and had 716 SNPs different, indicating that they were unrelated strains.

We could not find a single study on antimicrobial resistance genes in *Salmonella* from pigs in the Caribbean, and only few on *Salmonella* as a whole in the Caribbean. In poultry strains in Trinidad, a quite higher prevalence of resistance was found compared to our study, and this included plasmid-mediated colistin resistance [36,37,38].

All monophasic *S.* Typhimurium strains carried the IncQ1_1 plasmid. The full sequence of 6644 bp of this mobilizable plasmid was obtained. This plasmid also has been found in other *Salmonella* and *E. coli* (BLAST search 2/2021), as well as in *Aeromonas hydrophila* isolated from swine [39]. The IncI1 plasmid, found in one of the monophasic *S.* Typimurium strains, is a conjugative plasmid frequently associated with antimicrobial resistance genes, though here we could not identify any. However, since the sequence was not closed or complete (though it consisted of 68,689 bp), we could not confirm this. Nevertheless, we found genes associated with heavy metal resistance: there was a silver and copper resistance gene cluster on this plasmid. Copper is used in swine rearing, and this use might have selected for this resistance.

The two IncHI1A_1 and IncHI1B_1 sequences were found on the same contig, indicating this was a hybrid plasmid with two different replicons. Most likely, the IncFIA(HI1)_1_HI1 replicon was also on this plasmid, as this rep protein was on a single gene contig, and has been reported before on the same plasmid in other different serotypes of *Salmonella* worldwide [40]. This combination of replicons, together with an IncN replicon, has also been found in *E. coli*. This plasmid carried an *mcr*-gene encoding colistin resistance [41]. A plasmid with exactly the same sequences was found in *S.* Brandenburg and *S.* Ohio from two different farms, indicating its spread amongst *Salmonella* in Suriname. Using BLAST on the NCBI database, a similar plasmid, though with lower coverage and similarity, was found in a *Klebsiella* strain. Similarly, the ColpVC_1 plasmid was found worldwide in different bacterial species [42]. It is clear that all the col plasmids spread between the different Salmonella serotypes in Suriname.

It was somehow difficult to locate all resistance genes, as Illumina sequencing, also depending on the sequence depth, created several contigs that cannot be linked. Nevertheless, on bigger contigs as well, it is not always evident to link to structures. We could link the cluster encoding aminoglycoside, sulphomamide, and β-lactam (*aph(6)-Id* (*strB*), *aph(3″)-Ib* (*strA*), *sul2*, *bla*_TEM-1_) resistance to an Tn7-like structure, also indicating its mobility, as it was present in several strains and moving around as coresistances. This means when one of the antimicrobials was used, it selected for all the resistance genes, and thus created a larger problem. Moreover, we also had to take into account the use of heavy metals such as zinc and copper in veterinary medicine. Resistance genes against these metals are also located on this mobile genetic element (data not shown). The *aph(3′)-Ia* gene could not be linked to any mobile structure. The location of the *tet*(B) gene could not really be determined; however, through BLAST analysis, the same genes in the contig were also present in *Glasseralla parasuis*, *E. coli*, and *Shigella flexneri*, which indicated the mobility or preferential location of this gene.

## 5. Conclusions

In conclusion, this was the first study on the prevalence of *Salmonella* in pigs in the Caribbean. About 25 of all the farms in Suriname were sampled, and prevalence was 9% (CI 3.1–21%). Few strains were isolated, although they included the pandemic monophasic *S.* Typhimurium ST34. Fewer resistances were found compared to other monophasic *S.* Typhimurium ST34 strains isolated in other countries. In general, the strains were rather susceptible, except for aminoglycoside resistance. Most resistances could be located on mobile genetic elements, with a multi-resistant, Tn7-like element spreading. Local comparison of similar serotypes showed a rather clonal spread of certain serotypes. More strains should be analyzed to determine the local epidemiology of *Salmonella* in pigs in Suriname. The fact that no strains could be isolated from meat samples showed that food safety is not hampered very much by the presence of *Salmonella* in pigs.

## Figures and Tables

**Table 1:** Isolation and serotype results and origin of the strains.

Isolate	Farm	Origin	Serotype	MLST
173	B	Cecal	Anatum	ST64
174 *	X	Cecal	Ohio	ST329
175 *	X	Cecal	Monophasic variant of Typhimurium	ST34
179	X	Lymph node	Monophasic variant of Typhimurium	ST34
180	P	Lymph node	Monophasic variant of Typhimurium	ST34
222	E	Cecal	Brandenburg	ST65
250	X	Lymph node	Javaniana	ST1674
543	F	Lymph node	Ohio	ST329

* Strains from the same animal.

**Table 2:** T Antimicrobial resistance and plasmids associated with the different isolates.

Isolate	Serotype	β-Lactam	Aminoglycoside	Sulphonamide	Tetracycline	Trimethoprim	Plasmids
173	Anatum		*aac(6′)*				ColpVC_
174	Ohio		*aac(6′)*				
175	Monophasic variant of Typhimurium	*bla*_TEM-1_	*aac(6′), aph(6)-Id, aph(3* *″* *)-Ib, aph(3′)-Ia*	*sul2*			IncQ1_1
179	Monophasic variant of Typhimurium	*bla*_TEM-1_	*aac(6′), aph(6)-Id, aph(3* *″* *)-Ib, aph(3′)-Ia*	*sul2*			IncQ1_1, IncI1_1_Alpha
180	Monophasic variant of Typhimurium	*bla*_TEM-1_	*aac(6′), aph(6)-Id, aph(3* *″* *)-Ib, aph(3′)-Ia*	*sul2*			IncQ1_1
222	Brandenburg	*bla*_TEM-1_	*aac(6′), aph(6)-Id, aph(3* *″* *)-Ib*	*sul2*	*tet*(B)		IncFIA(HI1)_1_HI1, IncH1A/B, ColRNAI_1, Col440I_1
250	Javaniana		*aac(6′)*				
543	Ohio	*bla*_TEM-1_	*aac(6′), aph(6)-Id, aph(3* *″* *)-Ib*	*sul2*	*tet*(B)	*dfrAB*	IncFIA(HI1)_1_HI1, IncHI1B(R27)_1_R27, Col440I_1

## Data Availability

Raw sequences (fastq files) were submitted to the NCBI database under PRJNA751882 with SAMN20584910, SAMN20584911, SAMN20584912, SAMN20584913. SAMN20584914, SAMN20584915, SAMN20584916, and SAMN20584917.
